# Effect of a Healing Program Using Marine Resources on Reducing Pain and Improving Physical Function in Patients with Non-Specific Chronic Low Back Pain: A Randomized Controlled Trial Study

**DOI:** 10.3390/medicina61020172

**Published:** 2025-01-21

**Authors:** Ji-Eun Baek, Sung-Hyeon Kim, Ho-Jin Shin, Hwi-Young Cho

**Affiliations:** 1 Department of Physical Therapy, Gachon University, Incheon 21936, Republic of Korea; baekjieun421@gmail.com (J.-E.B.); gpgkorea30@gmail.com (S.-H.K.); 2 Wellness Center, Industry-University Collaboration Group, Ansan University, Ansan 15328, Republic of Korea; hojin0911@ansan.ac.kr

**Keywords:** non-specific chronic low back pain, marine therapy, physical health, psychological health

## Abstract

*Background and Objectives*: Chronic low back pain is a widespread condition, particularly in older populations, contributing to physical, mental, and social burdens. Traditional treatments, such as medications and surgery, carry long-term risks, including dependency, side-effects, and complications from invasive procedures. Additionally, healthcare accessibility is limited due to high costs, long waiting times, and geographic disparities in healthcare services, particularly in rural areas. For these reasons, non-pharmacological approaches that address both physical and psychological aspects are increasingly recognized as effective. This study aimed to evaluate the effectiveness of a marine resource-based healing program in Taean, South Korea, in improving pain, physical function, and mental health in patients with non-specific chronic low back pain. *Materials and Methods*: This randomized controlled trial involved 46 participants with non-specific chronic low back pain (mean age, 68.7 ± 5.1 years), randomly allocated to either an experimental group (marine healing program) or a control group (core exercises). The experimental group participated in a 4-night, 5-day intervention comprising heated peat pack therapy, mindfulness meditation, core exercises, and local tourism. The control group performed core exercises without additional interventions. Key outcomes included pain, muscle properties, functional disability, lower extremity function, balance, gait, and depression. *Results*: The experimental group demonstrated significant reductions in resting pain (*p* < 0.001), improved pressure pain threshold at L3 (*p* < 0.001), decreased L3 muscle tone (*p* = 0.015), and improved functional disability scores (*p* < 0.001). Functional gains were observed in lower extremity function scores (*p* < 0.001), balance (sway area: *p* = 0.046), gait velocity (*p* < 0.001), and depression levels (*p* < 0.001). *Conclusions*: The marine healing program significantly improved pain, functional abilities, and mental well-being in patients with non-specific chronic low back pain, highlighting its potential as an integrative approach to chronic low back pain management. Further studies are recommended to explore long-term and generalized effects.

## 1. Introduction

Back pain is a common health problem worldwide, and its prevalence is increasing, especially due to population aging and lifestyle changes [1,2,3,4]. Chronic low back pain (CLBP) continues to be defined as pain localized in the lumbar region, lasting for at least half the days in the past 6 months without an identifiable specific pathology [5]. It is particularly prevalent among women and individuals aged 40 to 80, but recent studies have highlighted a growing incidence among younger adults, driven by sedentary lifestyles, prolonged sitting, and reduced physical activity [6]. This trend underscores the need for proactive management across all age groups. However, older adults face distinct challenges, including a higher prevalence of comorbidities, reduced mobility, and greater vulnerability to the long-term risks associated with conventional treatments, such as medication dependency and surgical complications [7]. Furthermore, healthcare accessibility issues—particularly for rural or underserved populations—make non-pharmacological, integrative approaches more critical for this demographic. These approaches, which address both physical and psychosocial aspects, are vital for reducing the overall disease burden, improving patient outcomes, and lowering healthcare costs, especially in older adults with limited access to specialized care.

In particular, the management of non-specific chronic low back pain (NSLBP) has emerged as an important task in public health [8]. Because the pathoanatomical cause of NSLBP is unclear, a customized, non-pharmacological treatment approach that goes beyond pain relief to address psychosocial and lifestyle factors is required. Such management plays a vital role in reducing the overall burden of disease and improving patient outcomes by lowering healthcare costs and enhancing productivity.

Current approaches to managing low back pain have several limitations and challenges. Most treatments are hospital-based, limiting patient access, and drug treatments, in particular, carry the risk of developing resistance [9]. Additionally, surgery is considered a last resort for chronic low back pain, which increases the burden on patients and limits the sustainability of treatment. To address these issues, there is a growing need for safe and accessible alternative treatment methods. Heat therapy and exercise have been proposed as effective, low-risk alternatives. Heat application is widely used to relieve chronic musculoskeletal pain, while exercise plays a critical role in alleviating pain, improving muscle strength, and enhancing motor function in patients with NSLBP.

The integration of neuroscience-based pain education (PNE) with exercise therapy has demonstrated promising results in managing non-specific chronic low back pain (CLBP). PNE educates patients about the biological and physiological mechanisms of pain, fostering improved self-management and reducing fear-avoidance behaviors. When combined with neuromuscular or targeted exercise programs, this approach effectively alleviates pain intensity, reduces functional disability, and addresses psychological barriers such as kinesiophobia and catastrophizing beliefs. For instance, a study evaluating the effects of an eight-week PNE and neuromuscular exercise program reported significant improvements in pain, disability, and fear-avoidance behaviors compared to exercise alone [10]. Similarly, research indicates that combining PNE with motor control training yields greater reductions in central sensitization and psychological distress than standalone interventions [11]. Furthermore, a multimodal approach incorporating PNE, physical exercises, mindfulness, and behavior change strategies has been shown to enhance overall health outcomes and reduce reliance on primary healthcare services [12].

Additionally, previous studies have demonstrated the effectiveness of various physical and occupational therapy interventions for managing chronic low back pain, including core stabilization exercises, aquatic therapy, manual therapy, and psychosocial approaches such as cognitive-behavioral therapy (CBT) and yoga. These interventions have shown significant benefits in reducing pain, improving function, and enhancing mental well-being [13,14,15,16,17]. Nonetheless, clinical trials on interventions for chronic NSLBP remain insufficient [18].

The effective management of chronic back pain must go beyond physical treatment to comprehensively address the patient’s psychological, mental, and emotional state. Recent studies have demonstrated that these factors significantly influence the occurrence, persistence, and chronicity of low back pain, suggesting that psychosocial approaches, such as cognitive behavioral therapy, may be effective [19,20,21,22]. Thus, in the management of chronic low back pain, understanding and addressing psychological, mental, and emotional factors are as important as physical treatment, making the development and application of integrated treatment approaches essential.

Taean’s marine environment provides ideal conditions for managing chronic back pain, offering an abundance of marine resources and natural healing properties. This region is rich in healing resources, particularly peat, which is a decomposed organic material found in wetland environments. Rich in humic acids and minerals, peat possesses anti-inflammatory and thermotherapeutic properties, making it effective for managing musculoskeletal pain [23,24]. Additionally, Taean’s natural environment supports healing programs that leverage elements such as sunlight, the sound of waves, and fresh air, all of which contribute to improved physical and mental health [25,26]. Research has shown that visiting natural environments reduces depression and high blood pressure, viewing natural scenery alleviates pain and anxiety, and sunlight exposure is especially beneficial in improving mood [27,28]. Exercise in a marine environment like Taean can enhance mental health, while elements such as ocean sounds and sand have potential for use in natural healing programs [29,30,31]. Taean’s unique environment presents a novel direction in the utilization of natural healing resources for chronic pain management and can become a cornerstone of preventive medicine.

This study aims to contribute to the development of an integrative and sustainable non-pharmacological management approach for non-specific chronic low back pain (NSLBP), with a particular focus on utilizing marine resources. This novel intervention seeks to complement existing hospital-based treatments by addressing physical, psychological, and environmental factors.

The primary purpose of this study is to evaluate the effectiveness of a healing program utilizing marine resources for patients with non-specific chronic low back pain (NSLBP). This study hypothesizes that this marine healing program will be more effective than conventional core exercises in reducing pain, improving function, and promoting mental health.

## 2. Materials and Methods

### 2.1. Study Design

This study was conducted as a single-blinded, randomized controlled study. To maintain blinding, data were coded to ensure that the analyst was unaware of the participants’ group allocations.

### 2.2. Participants

Participants in this study were recruited through poster advertisements and cooperation with the Taean County Health and Medical Center located in Taean, Chungcheongnam-do. Among the volunteers who wanted to participate, those who reported a pain score of 3 or higher on the visual analog scale (VAS) at rest in the back and legs, had pain for more than 6 months, and required medication to relieve pain were recruited. The exclusion criteria were as follows: (1) individuals requiring other medical, pharmacological, or alternative treatments for back pain symptoms during the intervention period, (2) individuals with musculoskeletal conditions (including a history of surgery) or pain caused by identifiable neurological pathologies that could affect measurements, (3) individuals who had difficulty communicating in Korean, and (4) individuals at risk of mental health issues as indicated through self-report.

A total of 46 patients with back pain who met the criteria ultimately participated in the experiment. Before participating in this study, all subjects signed an informed consent form after receiving an explanation of the study. This study was conducted after receiving approval from the Gachon University Institutional Review Board (approval number: 1044396-202108-HR-181-01 and 9 October 2021) and was registered with the Clinical Research Information Service of the Korea Disease Control and Prevention Agency (registration number: KCT0009103).

### 2.3. Sample Size

The software G*Power 3.1.9.7 (Universität Kiel, Kiel, Germany) was used to calculate the sample size. According to the study by Faul et al., the effect size was set at 0.25, 80% power, and α = 0.05, requiring 34 participants. Considering a dropout rate of 20%, a total of 51 participants were recruited, and ultimately 46 participants completed this study.

### 2.4. Study Sites

The intervention in this study was divided into an experimental group and a control group.

The experimental group conducted activities at a training center located in Cheongpodae-gil, Nam-myeon, Taean-gun, Chungcheongnam-do, South Korea. The nearby Cheongpodae Beach consists of a dense pine forest and a wide sandy beach. The white sandy beach, composed of silica sand, spans an area of 30,000 m^2^, with a length of 1000 m and a width of 30 m. Its gentle slope of 6 degrees makes it suitable for beach trekking [32].

In contrast, the control group performed core exercises between their home and school without separate accommodation.

### 2.5. Procedure

**Figure 1:**
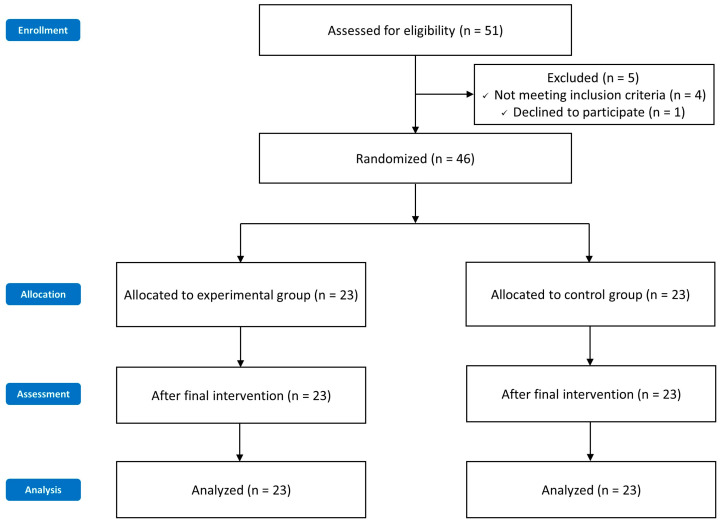
Flowchart.

Participants, selected after considering the inclusion and exclusion criteria, signed the consent form for participation in this study and completed pre-intervention measurements. They were then randomly assigned to one of two groups (experimental or control group) and participated in their respective interventions for 4 nights and 5 days. Random allocation in this study was performed using Microsoft Excel (Microsoft Corp., Redmond, WA, USA), where participants were randomly assigned to the experimental and control groups using blocks of size 4. Randomization was performed by a physical therapist who was blinded to the study hypothesis and group assignments. Post-measurements were conducted immediately after completing the interventions (Figure 1).

All interventions and measurements were conducted by licensed physical therapists with more than 3 years of clinical experience. These therapists were not involved in the recruitment of participants, to ensure objectivity.

### 2.6. Intervention

Participants performed the assigned interventions for 4 nights and 5 days (Table 1). The experimental group underwent a program involving heated peat packs, core exercises, mindfulness meditation, and local tourism, while the control group performed only core exercises in the city center.

A heated peat pack, maintained at 40 °C, was applied to the experimental group for 40 min on the area experiencing back pain. An electric heating pack ensured consistent heat conduction. Mindfulness meditation, focusing on breathing and bodily sensations, was provided to the experimental group as part of a marine healing program. Previous studies have shown that this type of intervention reduces stress and improves health [33,34]. Additionally, the experimental group participated in local tourism activities near Taean, including walking along the coast, visiting the Cheongsan Arboretum, attending the Taean Light Festival, and experiencing tidal flats. Local tours were conducted under researcher supervision to ensure participant safety.

The experimental and control groups both performed core exercise interventions. The 50 min sessions included a warm-up, main exercise, and cool-down. The warm-up and cool-down exercises focused on stretching and improving mobility, while the main exercise, lasting 30 min, involved core muscle function training, including abdominal breathing, bridge exercises, dead bug exercises, plank exercises, bird dog exercises, and side bridge exercises. Participants began with static exercises (e.g., abdominal breathing, planks) and progressed to dynamic exercises (e.g., bird dogs, side bridges). Each exercise was performed for 3 sets of 10 repetitions and was tailored to the individual abilities of participants. The difficulty was gradually increased as participants adapted to the exercises.

The control group received the same core exercises as the experimental group but did not participate in heated peat packs, mindfulness meditation, or local tourism.

To minimize potential biases related to human or seasonal factors, the same research team administered the interventions to both the experimental and control groups, ensuring consistency, with a one-week interval between the groups.

### 2.7. Outcome Measures

#### 2.7.1. Primary Outcome (Pain, Pressure Pain Threshold, Properties of Muscles, Disability)

The Visual Analog Scale (VAS) was used to determine participants’ pain levels. Participants were instructed to draw a straight line perpendicular to a line marked with 0 (no pain) and 10 (most severe pain imaginable) to indicate their level of back pain at rest and during activities such as housework or daily tasks. The test–retest reliability of this tool was found to be 0.97 [35].

The threshold for pressure-induced pain was measured using a digital algometer (PAIN TEST^TM^ FPIX, Greenwich, CT, USA). While lying prone in a comfortable position, participants indicated when they first experienced discomfort or pain as the evaluator applied increasing pressure 2 cm lateral to the spinous processes of the L3 and L5 vertebrae. The intra-rater reliability for this tool was 0.932 [36].

Muscle tone and stiffness were assessed using the Myoton (Myoton AS, Tallinn, Estonia) while participants lay prone. The tool was applied 2 cm lateral to the spinous processes of the L3 and L5 vertebrae. The intra-rater reliability of this tool was above 0.750 [37].

The Oswestry Disability Index (ODI) was used to evaluate functional disability due to back pain. This self-administered questionnaire consists of 10 questions scored on a 5-point scale. The total score is multiplied by 2 to generate a percentage, with higher scores indicating greater disability. The Cronbach’s α value of this tool was 0.73 [38].

#### 2.7.2. Secondary Outcome (Lower Extremity Function, Balance, Gait, Depression)

Lower extremity function was evaluated using the Short Physical Performance Battery (SPPB), which includes static balance, walking speed, and chair rise tests. The test–retest reliability of this tool was over 0.83 [39].

Balance ability was assessed using the Accusway force plate (Advanced Mechanical Technology, Inc., Watertown, MA, USA) by measuring shifts in the center of gravity during standing with eyes open and closed. Participants stood with their heels approximately 9 cm apart and arms crossed in front of their chest and focused on an X-shaped target at eye level. The reliability of this tool was above 0.7 [40].

Gait ability was measured using the GAITRite walkway (CIR Systems Inc., Franklin, NJ, USA). Spatiotemporal walking variables were calculated from center of pressure (COP) data obtained using sensors embedded in the mat. Measurements were conducted in a disturbance-free environment, with participants instructed to walk at a comfortable speed. The reliability of this tool was above 0.92 [41].

Depression severity was assessed using the Beck Depression Inventory (BDI), a self-report questionnaire with 21 items. Scores were categorized as follows: 0–9 (no or minimal depression), 10–18 (mild to moderate depression), 19–29 (moderate to severe depression), and 30–63 (very severe depression). The Cronbach’s α value of this tool was 0.89 [42].

### 2.8. Statistical Analysis

Data collected in this study were analyzed using SPSS version 25.0 (IBM Corp., Armonk, NY, USA). The normality of the data was confirmed using the Shapiro–Wilk test. Homogeneity between groups was assessed using independent *t*-tests and chi-squared (χ^2^) tests. Differences within and between groups were analyzed using mixed repeated-measures analysis of variance (ANOVA). Tukey’s HSD test was used for post hoc testing. Statistical significance was set at α = 0.05.

## 3. Results

### 3.1. General Characteristics of Participants

All participants completed the program without dropping out. The demographic characteristics of the participants in this study are as follows (Table 2).

### 3.2. Primary Outcomes

The primary outcomes in this study were as follows (Table 3).

#### 3.2.1. Pain

Pain at rest and pain during movement showed significant improvement in both the experimental and control groups after the intervention, and in particular, the experimental group showed greater improvement than the control group (*p* < 0.05).

#### 3.2.2. Pressure Pain Threshold

The pressure pain threshold at L3 and L5 levels was significantly improved in the experimental group after intervention (*p* < 0.05). On the other hand, no significant changes were observed in the control group (*p* > 0.05).

#### 3.2.3. Properties of Muscles

Among muscle properties, tone and stiffness at the L3 level and tone at the L5 level were significantly improved in the experimental group after intervention (*p* < 0.05). However, in the case of stiffness at the L5 level, no significant improvement was observed in both the experimental and control groups (*p* > 0.05). There was no change in muscle properties in the control group (*p* > 0.05).

#### 3.2.4. Disability

In the case of disability level, significant improvement was observed in the experimental group after intervention (*p* < 0.05). There was no significant improvement in the control group (*p* > 0.05).

### 3.3. Secondary Outcomes

The secondary outcomes in this study were as follows (Table 4).

#### 3.3.1. Lower Extremity Function

Lower extremity function showed significant improvement in the experimental group after intervention (*p* < 0.05). No significant changes were observed in the control group (*p* > 0.05).

#### 3.3.2. Balance

In terms of balance ability, the sway area showed significant improvement in the experimental group after intervention (*p* < 0.05). However, in the case of sway velocity, no significant changes were observed in all groups (*p* > 0.05).

#### 3.3.3. Gait

The experimental group showed significant improvement in all variables of walking ability after intervention (*p* < 0.05). The control group showed no significant changes (*p* > 0.05).

#### 3.3.4. Depression

There was significant improvement in the level of depression in the experimental group after intervention (*p* < 0.05). There was no significant change in the control group (*p* > 0.05).

## 4. Discussion

In this study, we found that a natural healing program using Taean’s marine resources was effective in reducing pain, decreasing muscle tension, and improving daily living skills in patients with non-specific chronic low back pain (NSLBP). These results suggest that marine healing programs can contribute to improving both physical and mental health.

Visiting natural environments can play an important role in pain management, as it has been shown to increase the pain threshold and tolerance [43]. The results of this study are consistent with previous findings that demonstrated positive effects of meditation and exercise in marine areas on various health aspects, including pain intensity, tactile spatial accuracy, balance, overall quality of life, and depression scores [44].

This study suggests that the effects of the marine healing program on pain reduction and functional improvement in patients with non-specific chronic low back pain (NSCLBP) may be attributed to its psychological and mental benefits. Chronic pain patients often experience anxiety, depression, and cognitive impairments, which are associated with structural and functional changes in the brain, particularly in gray matter volume [45]. These neurological changes affect regions involved in pain processing, emotional regulation, and cognition. Notably, individuals with chronic low back pain (CLBP) have been reported to struggle with emotional processing [20,21]. Previous studies have highlighted the effectiveness of psychosocial approaches such as CBT, yoga, and relaxation techniques in reducing pain perception and improving overall quality of life for chronic pain patients [46,47]. Environmental factors, lifestyle modifications, and mind–body practices such as yoga and meditation have been shown to mitigate pain perception and counteract brain changes associated with chronic pain [45]. Furthermore, nature-based meditation has demonstrated significant effects in enhancing psychological and physiological well-being, fostering interpersonal relationships, increasing positive emotions, and reducing negative emotions [48]. Marine healing programs, in particular, promote emotional stability and alleviate stress, which may contribute to reducing low back pain and improving functional outcomes [49]. These interventions address the psychological factors associated with pain chronicity, similar to the benefits observed in the marine healing program. Moreover, the program’s unique combination of peat therapy, mindfulness meditation, and natural environmental exposure enhances emotional stability and reduces stress, contributing to pain alleviation and functional improvement. Taean’s marine resources, including coastal scenery and fresh air, further amplify these benefits, aligning with findings that natural environments positively influence physical and mental health [50,51,52]. The findings of this study support the conclusion that marine healing programs enhance participants’ emotional processing abilities, thereby contributing to the reduction in low back pain.

Marine therapy, encompassing marine-derived treatments such as thalassotherapy and algotherapy, has demonstrated substantial benefits in managing various diseases due to the bioactive compounds in marine resources. Thalassotherapy, involving seawater, sand, and marine products, is known for its anti-inflammatory and immunomodulatory effects, particularly in dermatological conditions like psoriasis, eczema, and vitiligo, as well as rheumatologic disorders such as fibromyalgia and rheumatoid arthritis [53]. Algae, central to algotherapy, contain high levels of vitamins, amino acids, and minerals that aid in cardiovascular health, metabolic regulation, and skin repair [54]. Seawater’s mineral-rich composition further supports wound healing, antioxidant activity, and disease management [55].

Core exercises are widely recognized for their ability to strengthen trunk muscles and enhance lumbar stability, which can contribute to pain relief and improved spinal support [56,57]. By strengthening the core, these exercises help alleviate strain on the spine and promote a better balance of the muscles surrounding the spinal column [58]. As a result, core exercises may have a positive impact on improving individuals’ ability to carry out daily activities by reducing pain, improving static balance, and enhancing lower extremity function and walking ability. In the program conducted as part of this study, participants engaged in core exercises daily throughout the intervention period. Although the program lasted only five days, the benefits of these exercises likely contributed to the reduction in participants’ low back pain. Although muscle strengthening typically requires sustained exercise programs over weeks or months, short-term interventions can transiently reduce muscle tone and stiffness through increased circulation and neuromuscular activation [24]. This aligns with the observed improvements in muscle tone after 5 days of basic exercises.

The results of this study demonstrate the significant potential of Taean’s marine resources for enhancing both physical and mental health. The natural environment of this region offers benefits such as reducing inflammation and pain, while the marine elements, in combination with exercise and healing programs, contribute to improved mental well-being [25,26,27]. Previous studies have similarly highlighted the benefits of natural environments for alleviating depression and hypertension, with sunlight exposure playing a particularly effective role in improving depressive symptoms [28]. These elements of the marine environment can play a vital role in chronic pain management and preventive healthcare. Taean’s marine resource healing program, by harnessing these natural elements, presents a promising approach to health promotion. Moreover, this study aligns with the principles outlined in the latest clinical practice guidelines, including those from the American College of Physicians (ACP) and the National Institute for Health and Care Excellence (NICE) [7,59]. By integrating evidence-based exercise therapy with innovative interventions like marine healing programs, the findings highlight the potential for advancing noninvasive and patient-centered care for chronic low back pain. These results reinforce the conclusion that Taean’s marine resource healing program can significantly contribute to health promotion. While this study clarified the various positive effects of the marine healing program on patients with non-specific chronic low back pain, several limitations warrant caution in generalizing the findings. First, the study involved a limited number of participants, and the sample was drawn from a specific region, restricting the generalizability of the results. Future studies should aim to include a larger and more diverse population to enhance the scope and applicability of the findings. Second, the short duration of the study (4 nights and 5 days) limits the ability to comprehensively assess the program’s long-term benefits. Longitudinal studies with extended follow-up periods are essential, particularly to determine the persistence of physical gains over time and to evaluate the broader effects on functional outcomes. Third, the pressure pain threshold (PPT) measurements were limited to symptomatic areas (L3 and L5). While this approach allowed for a focused evaluation of the intervention’s localized effects on pain sensitivity and muscle properties, it did not account for potential systemic effects that could be observed by assessing PPT at distant, asymptomatic regions. Future studies should consider incorporating PPT measurements at both symptomatic and distant sites to provide a more comprehensive understanding of the intervention’s impact. Finally, comparative studies with other healing programs are needed to assess the relative effects of the marine healing program. The synergistic effects of the marine healing program and exercise likely contributed to the observed outcomes. While the psychological benefits of the marine environment, such as stress reduction and emotional regulation, enhance physical recovery, further studies are needed to evaluate the standalone efficacy of the marine healing program compared to exercise alone. Addressing these limitations through more comprehensive and rigorous study designs will allow for a more precise evaluation of the program’s effectiveness.

## 5. Conclusions

A marine healing program utilizing Taean’s marine resources is effective in reducing pain, alleviating muscle tension, and improving daily living skills in patients with non-specific chronic low back pain (NSLBP). This integrative approach addresses the limitations of conventional treatments by incorporating natural environmental benefits, physical therapy, and mindfulness practices. Future studies should focus on evaluating the program’s long-term effects and comparing its efficacy with other established interventions. Expanding the participant pool and diversifying demographics will also provide more generalizable results.

## Figures and Tables

**Table 1:** Schedule of the experimental group.

Time	Day 1	Day 2	Day 3	Day 4	Day 5
07:00–08:00		Coastal walk	Coastal walk	Coastal walk	Coastal walk
08:00–09:00		Breakfast	Breakfast	Breakfast	Breakfast
09:00–10:00		CORE exercise	CORE exercise	CORE exercise	CORE exercise
10:00–11:00		Mindfulness meditation	Mindfulness meditation	Mindfulness meditation	Mindfulness meditation
11:00–12:00		Break time	Break time	Break time	Post-measurement
12:00–13:00		Lunch	Lunch	Lunch	
13:00–14:00	Orientation	CORE exercise	CORE exercise	CORE exercise	Lunch
14:00–15:00	Pre-measurement	Nordic walking	Visiting Cheongsan Arboretum	Tideland activity	Check-out
15:00–16:00					
16:00–17:00	CORE exercise	Heated peat pack treatment	Heated peat pack treatment	Heated peat pack treatment	
17:00–18:00	Heated peat pack treatment	Break time	Break time	Dinner	
18:00–19:00	Dinner	Dinner	Dinner	Visiting Taean Light Festival	
19:00–20:00	Individual marine healing activities	Individual marine healing activities	Individual marine healing activities		
20:00–21:00	Washing and personal care				
21:00–22:00	Sleeping				

**Table 2:** General characteristics.

Variables	G1	G2	*p*-Value
Age (y) ^a^	68.91 ± 5.07	68.57 ± 5.05	0.817 ^†^
Sex (M/F) ^b^	7/16	8/15	0.753 ^‡^
Height (cm) ^a^	158.24 ± 7.65	158.46 ± 7.62	0.924 ^†^
Weight (kg) ^a^	62.23 ± 9.69	61.89 ± 9.43	0.905 ^†^
BMI (kg/m^2^) ^a^	24.80 ± 3.00	24.61 ± 2.92	0.823 ^†^
Percent body fat (%) ^a^	33.24 ± 6.82	34.26 ± 6.20	0.599 ^†^
Skeletal muscle mass (kg) ^a^	22.38 ± 4.58	24.25 ± 5.05	0.196 ^†^

^a^ Values are expressed as the mean ± SD. ^b^ Values are expressed as numbers. ^†^ Independent *t*-test used for comparison of continuous variables. ^‡^ Chi-squared test used for comparison of categorical variables. BMI, body mass index.

**Table 3:** Primary outcomes.

Variables	Group	Pre-Test	Post-Test	*p*-Value	ANOVA	η^2^_p_
		Mean ± SD	Mean ± SD			
PPT						
L3	G1	6.09 ± 2.72	7.76 ± 2.70	<0.001		
	G2	6.12 ± 2.61	6.19 ± 2.17	0.756	<0.001	0.34
	*p*-value	0.968	0.035			
L5	G1	5.73 ± 3.10	6.91 ± 3.16	0.030		
	G2	6.05 ± 3.16	5.64 ± 2.32	0.305	0.017	0.12
	*p*-value	0.725	0.127			
VAS						
Resting pain	G1	4.11 ± 1.75	1.17 ± 1.45	<0.001		
	G2	3.89 ± 1.70	3.40 ± 1.42	0.017	<0.001	0.39
	*p*-value	0.663	<0.001			
Movement pain	G1	5.74 ± 1.98	1.68 ± 1.61	<0.001		
	G2	5.47 ± 1.99	4.74 ± 1.61	0.010	<0.001	0.38
	*p*-value	0.645	<0.001			
Myoton						
L3 Tone	G1	19.00 ± 4.24	17.19 ± 2.30	0.015		
	G2	19.62 ± 4.32	19.34 ± 4.02	0.286	0.042	0.09
	*p*-value	0.625	0.031			
L3 Stiffness	G1	402.48 ± 112.61	357.00 ± 61.51	0.017		
	G2	377.79 ± 90.84	376.45 ± 91.35	0.372	0.016	0.12
	*p*-value	0.418	0.402			
L5 Tone	G1	17.19 ± 2.69	16.23 ± 4.04	0.045		
	G2	16.30 ± 3.06	16.57 ± 3.22	0.438	0.036	0.10
	*p*-value	0.300	0.751			
L5 Stiffness	G1	374.74 ± 93.14	350.87 ± 89.78	0.058		
	G2	340.83 ± 95.67	341.87 ± 98.30	0.900	0.092	0.06
	*p*-value	0.230	0.747			
ODI	G1	42.06 ± 12.61	24.72 ± 9.81	<0.001		
	G2	43.67 ± 13.84	40.72 ± 11.37	0.141	<0.001	0.28
	*p*-value	0.682	<0.001			

η^2^_p_, partial eta squared. PPT, pain pressure threshold; L, lumbar spine; VAS, visual analog scale; ODI, Oswestry Disability Index.

**Table 4:** Secondary outcomes.

Variables	Group	Pre-Test	Post-Test	*p*-Value	ANOVA	η^2^_p_
		Mean ± SD	Mean ± SD			
SPPB	G1	9.46 ± 1.74	10.96 ± 1.11	<0.001		
	G2	9.63 ± 1.37	9.72 ± 1.36	0.539	<0.001	0.35
	*p*-value	0.709	0.002			
BDI	G1	23.61 ± 12.36	6.74 ± 4.71	<0.001		
	G2	22.14 ± 11.56	21.90 ± 11.81	0.336	<0.001	0.47
	*p*-value	0.679	<0.001			
Balance ability						
Sway area (cm^2^)	G1	289.55 ± 230.78	208.80 ± 143.03	0.046		
	G2	303.28 ± 238.40	316.46 ± 267.96	0.182	0.021	0.11
	*p*-value	0.844	0.096			
Sway velocity (cm/s)	G1	15.87 ± 8.24	13.96 ± 7.16	0.242		
	G2	16.71 ± 8.71	16.80 ± 7.94	0.901	0.256	0.03
	*p*-value	0.738	0.209			
Gait ability						
Velocity (cm/s)	G1	110.70 ± 13.09	122.84 ± 12.67	<0.001		
	G2	104.56 ± 14.16	106.86 ± 26.45	0.636	0.065	0.08
	*p*-value	0.134	0.012			
Cadence (step/min)	G1	109.27 ± 6.91	113.41 ± 7.63	<0.001		
	G2	103.21 ± 9.77	100.24 ± 15.86	0.355	0.036	0.10
	*p*-value	0.019	<0.001			
Step length (cm)	G1	60.68 ± 6.87	64.24 ± 8.58	0.020		
	G2	64.01 ± 8.57	63.97 ± 7.34	0.971	0.048	0.09
	*p*-value	0.153	0.911			

η^2^_p_, partial eta squared; SPPB, Short Physical Performance Battery; BDI, Beck Depression Inventory.

## Data Availability

The data used in this study are available from the corresponding author upon request but are not publicly accessible, due to ethical restrictions.
